# Refined understanding of the impact of the *Mycobacterium tuberculosis* complex diversity on the intrinsic susceptibility to pretomanid

**DOI:** 10.1128/spectrum.00070-24

**Published:** 2024-02-09

**Authors:** Praharshinie Rupasinghe, Rabab Reenaers, Jens Vereecken, Wim Mulders, Sari Cogneau, Matthias Merker, Stefan Niemann, Shaheed Vally Omar, Leen Rigouts, Claudio U. Köser, Tom Decroo, Bouke C. de Jong

**Affiliations:** 1 Unit of Mycobacteriology, Department of Biomedical Sciences, Institute of Tropical Medicine, Antwerp, Belgium; 2 Department of Biomedical Sciences, University of Antwerp, Antwerp, Belgium; 3 Molecular and Experimental Mycobacteriology, Research Center Borstel, Borstel, Germany; 4 Evolution of the Resistome, Research Center Borstel, Borstel, Germany; 5 German Center for Infection Research, Partner site Hamburg-Lübeck-Borstel-Riems, Parkallee, Borstel, Germany; 6 Center for Tuberculosis, National Institute of Communicable Diseases, a division of the National Health Laboratory Service, Johannesburg, South Africa; 7 Department of Genetics, University of Cambridge, Cambridge, United Kingdom; 8 Unit of HIV and TB, Department of Clinical Sciences, Institute of Tropical Medicine, Antwerp, Belgium; Foundation for Innovative New Diagnostics, Geneve, Switzerland

**Keywords:** pretomanid, MTBC lineages, *Mycobacterium tuberculosis*

## Abstract

Previous work reported unprecedented differences in the intrinsic *in vitro* susceptibility of the *Mycobacterium tuberculosis* complex (MTBC) to pretomanid (Pa) using the Mycobacteria Growth Indicator Tube (MGIT) system. We tested 125 phylogenetically diverse strains from all known MTBC lineages (1–9) without known Pa resistance mutations and four strains with known resistance mutations as controls. This confirmed that MTBC, unlike most bacteria-antimicrobial combinations, displayed substantial differences in the intrinsic susceptibility relative to the technical variation of Pa MIC testing. This was also the case for the Middlebrook 7H11 (7H11) medium, demonstrating that these differences were not specific to MGIT. Notably, lineage 1 was confirmed to have intrinsically elevated MICs compared with lineages 2, 3, 4, and 7 (L2–4/7), underlining the urgent need for WHO to publish its decision of whether lineage 1 should be deemed treatable by BPaL(M), the now preferred all-oral regimen for treating rifampin-resistant tuberculosis. Lineages 5 and 6, which are most frequent in West Africa, responded differently to Pa, with lineage 5 being more similar to L2–4/7 and lineage 6 being more susceptible. More data are needed to determine whether 7H11 MICs are systematically lower than those in MGIT.

**IMPORTANCE:**

This study confirmed that the *Mycobacterium tuberculosis* complex lineage 1, responsible for 28% of global tuberculosis cases, is less susceptible to pretomanid (Pa). It also refined the understanding of the intrinsic susceptibilities of lineages 5 and 6, most frequent in West Africa, and lineages 8 and 9. Regulators must review whether these *in vitro* differences affect the clinical efficacy of the WHO-recommended BPaL(M) regimen and set breakpoints for antimicrobial susceptibility testing accordingly. Notably, regulators should provide detailed justifications for their decisions to facilitate public scrutiny.

## INTRODUCTION

The all-oral BPaL(M) regimen, consisting of bedaquiline, pretomanid (Pa), linezolid, and moxifloxacin (moxifloxacin is stopped if fluoroquinolone resistance is detected), is becoming the preferred option for treating rifampin-resistant tuberculosis (TB) (1, 2). Pa poses two challenges in this context. First, Bateson et al. (3) described unprecedented differences in the intrinsic susceptibility of different *Mycobacterium tuberculosis* complex (MTBC) lineages to Pa using the Mycobacteria Growth Indicator Tube (MGIT) system. Most notably, lineage 1 (L1), which accounts for 28% of TB cases globally, was found to be intrinsically less susceptible than the other major MTBC lineages [lineage 2 (L2), lineage 3 (L3), and lineage 4 (L4)], raising the question whether L1 responds equally well to BPaL(M) compared with L2–4 (3).

Second, clinical strains with high Pa MICs due to mutations in known Pa resistance genes were identified without known nitroimidazole exposure, suggesting genetic drift or yet unknown selective pressures (3–5). In the few settings with good surveillance or routine antimicrobial susceptibility (AST) results, these mutants are rare (5, 6). However, because these mutants are known to be transmissible, it is plausible that some settings exist in which an intrinsically Pa-resistant cluster is frequent, underlining the need for routine AST (4, 7). Yet, rapid AST directly from clinical samples is currently impossible as no commercial genotypic AST assay exists that interrogates *ddn* (*Rv3547*), *fbiA* (*Rv3261*), *fbiB* (*Rv3262*), *fbiC* (*Rv1173*), *fbiD* (*Rv2983*), and *fgd1* (*Rv0407*), the six genes required for the activation of the pro-drug Pa [no resistance mutations have been described in *dprE2* (*Rv3791*)], the target of Pa, to date) (8–10). Although efforts are underway to address this diagnostic gap (e.g., Genoscreen is evaluating Deeplex Myc-TB XL, an updated version of its WHO-endorsed targeted next-generation sequencing assay), the interpretation of genotypic AST results will remain a persistent challenge as the aforementioned resistance genes are non-essential and, consequently, thousands of different loss-of-function mutations can theoretically confer resistance (9, 11, 12).

The goal of this study was twofold. First, we used MGIT to refine the current understanding of the effect of the MTBC diversity on susceptibility, with a particular focus on the less frequent lineage 5 (L5), lineage 6 (L6), lineage 7 (L7), lineage 8 (L8), and lineage 9 (L9) that were not tested or were underrepresented in the literature (3, 13, 14). Second, we used Middlebrook 7H11 (7H11) as an alternative medium to investigate whether the differences observed with MGIT were media specific.

## MATERIALS AND METHODS

### Strains

We tested 125 MTBC strains from L1–9 from patients who had never received nitroimidazole treatment originating from 45 different countries on five different continents. Of these, 118 lacked known resistance mutations in the six canonical Pa-resistance genes and the remaining seven did not have whole-genome sequencing data but were selected based only on the treatment naivety to nitroimidazoles to augment L5 and L6. Of 125 strains, 49% (*n* = 61) were drug-susceptible (DS), 27% (*n* = 34) were mono-/poly-resistant (mono/PDR) to other TB drugs other than Pa, 23% (*n* = 29) were multidrug-resistant (MDR), and 1% (*n* = 1) was pre-extensively drug-resistant (Table S1) (15). All 125 strains were tested on 7H11, whereas a subset of 41 isolates were tested in MGIT. In addition, four Pa-resistant strains with known resistance mutations were included for both methods (Table 1). Of the total 129 strains, 10 strains were also tested by Bateson et al. (Table S2) (3). As per the ITM-IRB consultation, the fully anonymized use of clinical isolates for test validation did not require ethical review.

**TABLE 1:** MICs for Pa-resistant strains^*d*^

Strain ID	Lineage	DR profile	Genome accession	Pa resistance mutation		Study	Pa MIC (µg/mL)	
				*ddn*	*fbiC*		7H11	MGIT
2013-02481^*a*^	1.1.3	PDR	ERR8025345		Arg536Leu	Current	>8	>4
						Bateson et al.	NT	>16
2020-00011	1.1.3	MDR	ERR12115304	Trp27Stop^*c*^		Current	>8	>4
						Bateson et al.	NT	NT
2020-03565	1.1.3	DS	SAMN11179707	Trp27Stop^*c*^		Current	>8	>4
						Bateson et al.	NT	>8
2020-03568^*b*^	2.2.1	DS	ERR7361928	Gln58Stop^*c*^		Current	>8	>4
						Bateson et al.	NT	>16

^*a*^ *In vitro* mutant.

^*b*^ Selected by WHO as a resistant control strain for delamanid and Pa in the forthcoming AST manual.

^*c*^ Recognized as conferring cross-resistance to delamanid and Pa in the second edition of the WHO mutation catalogue (12).

^*d*^ NT, not tested.

### 7H11 MIC testing

Pa powder (Sigma-Aldrich SML-1290) was dissolved in dimethyl sulfoxide (DMSO) (Sigma D5879) to prepare a stock solution of 4,000 µg/mL and stored in 600 µL aliquots at −80°C. Standard 7H11 base was supplemented with 10% (vol/vol) oleic acid-albumin-dextrose-casein (OADC) enrichment and 0.5% (vol/vol) glycerol to prepare the 7H11 solid medium. A twofold dilution series of Pa ranging from 1 to 0.002 µg/mL plus 0.75 µg/mL (i.e., 11 concentrations in total) were tested for all strains, except for the four Pa-resistant strains, for which 0.25–8 µg/mL were used instead. Bacterial colonies were scraped from Löwenstein-Jensen slants and thoroughly homogenized in sterile water with glass beads. The density of the suspension was adjusted visually to McFarland 1. The least diluted growth control (GC1) and the drug-containing media in polypropylene tubes were inoculated with a 10^−1^ dilution of the McFarland 1 suspension, while the most diluted growth control (GC2) was inoculated with a 10^−3^ dilution. Colony-forming units (CFU) were enumerated after 4 weeks of incubation at 34°C–38°C with 5%–10% CO_2_. If both growth controls had sufficient growth at this point [i.e., at least 1+ (51–100 CFUs) on GC1 and 3 CFUs on GC2], CFU counts were recorded accordingly, and MIC results were interpreted. If GC1 and/or GC2 had insufficient CFUs at 4 weeks, tubes were incubated for two more weeks. Any test with insufficient CFUs on GC1 and/or GC2 after 6 weeks of incubation or more than 1+ growth on the GC2 was considered invalid and repeated once. The MIC was defined as the lowest drug concentration that inhibits the growth of more than 99% of the MTBC population.

Since MIC testing of Pa on 7H11 had not yet been established in our laboratory at the Unit of Mycobacteriology, Institute of Tropical Medicine, we first tested 30 replicates of the pan-susceptible H37Rv reference strain (BCCM/ITM CT2008-03715/ITM500735), using three different batches of Pa-containing medium, with repeated testing on different days over 10 weeks. Subsequently, H37Rv was included as a control in every batch of clinical strains.

### MGIT MIC testing

Pa working solutions prepared from the same stock solution used for 7H11 testing were added to MGIT tubes (100 µL each) to achieve 10 twofold Pa dilutions from 0.002 to 1 µg/mL, whereas higher concentrations (0.25–4 µg/mL) were tested for Pa-resistant strains. An inoculum was prepared directly from a positive MGIT tube that had flagged within 1–2 days or after a one in five dilution of a positive MGIT tube that had flagged within 3–5 days, and 500 µL of inoculum was added to the Pa-containing tubes supplemented with 800 µL of OADC. The drug-free control vial was inoculated with a 1:100 dilution of the inoculum. MICs were determined using MGIT 960 TBeXIST extended protocol according to the manufacturer’s instructions. The MIC was determined to be the lowest concentration at which the growth value of the drug-containing tube was <100 when the growth control had reached 400 growth units. A test resulting in an invalid code (×200 or ×400) was repeated once. Based on Bateson et al. (3), 0.06–0.5 µg/mL was used as a tentative quality control (QC) range, and a corresponding QC target of 0.125–0.25 µg/mL for H37Rv, which was included in every batch of clinical strains.

## RESULTS

### Technical reproducibility of Pa MIC testing

A good technical reproducibility was observed for both methods. Indeed, Pa MICs in MGIT were 0.125–0.25 µg/mL for H37Rv, corresponding to the tentative QC target (Table 2) (3). The corresponding 7H11 MICs were 0.06–0.125 µg/mL (no tentative QC range/target was available for comparison).

**TABLE 2:** MICs for H37Rv and clinical strains without known Pa resistance mutations^*g*^

Lineage	Medium	Study	Pa MIC range (µg/mL)	7H11 MICs (µg/mL)														Total
				Invalid	≤0.002	0.004	0.008	0.016	0.03	0.06	0.125	0.25	0.5	0.75	1	2	4	
1^*a*^	7H11	Current	0.25–1	–	–	–	–	–	–	–	–	4	**9**	6	2	NT	NT	21
	MGIT	Current	0.5–1	–	–	–	–	–	–	–	–	–	1	NT	**4**	NT	NT	5
		Bateson et al.	0.125–2	–	NT	NT	NT	–	–	–	2	3	39	NT	**76**	7	–	127
2^*b*^	7H11	Current	0.03–0.25	–	–	–	–	–	2	**21**	5	2	–	–	–	NT	NT	30
	MGIT	Current	0.125–0.25	–	–	–	–	–	–	–	1	**3**	–	NT	–	NT	NT	4
		Bateson et al.	0.06–1	–	NT	NT	NT	–	–	1	22	**24**	15	NT	1	NT	NT	63
3	7H11	Current	0.06–0.125	–	–	–	–	–	2	**6**	3	–	–	–	–	NT	NT	11
	MGIT	Current	0.125	–	–	–	–	–	–	–	**5**	–	–	NT	–	NT	NT	5
		Bateson et al.	0.06–0.125	–	–	NT	NT	–	–	4	**8**	–	–	NT	–	NT	NT	12
4^*c*^	7H11	Current	0.03–0.25	–	–	–	–	–	**17**	15	5	1	–	–	–	NT	NT	38
	MGIT	Current	0.125–0.25	–	–	–	–	–	–	–	**5**	2	–	NT	–	NT	NT	7
		Bateson et al.	0.03–0.5	–	–	–	–	–	3	25	**52**	31	2	NT	–	NT	NT	113
4-H37Rv^*d*^	7H11	Current	0.06–0.125	–	–	–	–	–	–	2	**36**	–	–	–	–	NT	NT	38
	MGIT	Current	0.125–0.25	–	–	–	–	–	–	–	2	**3**	–	NT	–	NT	NT	5
		Bateson et al.	0.06–0.5	–	NT	NT	NT	–	–	8^*e*^	47^*e*^^,*f*^	**60^*e*^** ^,*f*^	2^*e*^	NT	NT	NT	NT	117
5	7H11	Current	0.03–0.125	–	–	–	–	–	4	**6**	1	–	–	–	–	NT	NT	11
	MGIT	Current	0.03–0.06	–	–	–	–	–	2	**6**	–	–	–	NT	–	NT	NT	8
		Bateson et al.	0.03–0.06	–	NT	NT	NT	–	**1**	**1**	–	–	–	NT	–	NT	NT	2
6	7H11	Current	≤0.002–0.004	1	1	**9**	–	–	–	–	–	–	–	–	–	NT	NT	11
	MGIT	Current	≤0.002–0.008	–	1	**7**	1	–	–	–	–	–	–	NT	–	NT	NT	9
		Bateson et al.	0.004–0.016	–	–	1	1	1	–	–	–	–	–	NT	–	NT	NT	3
7^*c*^	7H11	Current	0.125	–	–	–	–	–	–	–	**1**	–	–	–	–	NT	NT	1
	MGIT	Current	0.125	–	–	–	–	–	–	–	**1**	–	–	NT	–	NT	NT	1
		Bateson et al.	0.25	–	NT	NT	NT	–	–	–	–	**3**	–	NT	–	NT	NT	3
8	7H11	Current	0.25	–	–	–	–	–	–	–	–	**1**	–	–	–	NT	NT	1
	MGIT	Current	1.0	–	–	–	–	–	–	–	–	–	–	NT	1	NT	NT	1
		Bateson et al.	NT															0
9	7H11	Current		1	–	–	–	–	–	–	–	–	–	–	–	NT	NT	0
	MGIT	Current	≤0.002	**–**	**1**	–	–	–	–	–	–	–	–	NT	–	NT	NT	1
		Bateson et al.	NT															0
*Mycobacterium bovis*	7H11	Current	NT															0
	MGIT	Current	NT															0
		Bateson et al.	0.03	–	NT	NT	NT	–	**3**	–	–	–	–	NT	–	NT	NT	3
*Mycobacterium caprae*	7H11	Current	NT															0
	MGIT	Current	NT															0
		Bateson et al.	0.03	–	NT	NT	NT	–	**1**	–	–	–	–	NT	–	NT	NT	1
*Mycobacterium microti*	7H11	Current	NT															0
	MGIT	Current	NT															0
		Bateson et al.	0.125	–	NT	NT	NT	–	–	–	**1**	–	–	NT	–	NT	NT	1
*Mycobacterium pinnipedii*	7H11	Current	NT															0
	MGIT	Current	NT															0
		Bateson et al.	0.06	–	NT	NT	NT	–	–	**1**	–	–	–	NT	–	NT	NT	1
*Mycobacterium canetti*	7H11	Current	NT															0
	MGIT	Current	NT															0
		Bateson et al.	2–4	–	NT	NT	NT	–	–	–	–	–	–	NT	–	**18**	3	21

^*a*^ Two strains were tested in both studies (Table S2).

^*b*^ Three strains were tested in both studies (Table S2).

^*c*^ One strain was tested in both studies (Table S2).

^*d*^ Replicates of H37Rv.

^*e*^ Tentative QC range based on Bateson et al. (3).

^*f*^ Tentative QC target based on Bateson et al. (3).

^*g*^ NT, not tested. Modes of MIC distributions are highlighted in bold text.

### MICs for strains with Pa resistance mutations

The four strains with known Pa resistance mutations had MICs of >4 µg/mL in MGIT, which was in line with earlier results, and >8 µg/mL using 7H11 (Table 1) (3).

### MICs for strains without Pa resistance mutations

Bateson et al. (3) reported a mode of 1 µg/mL and 99th percentile of 2 µg/mL for L1 MICs in MGIT, which was elevated compared with the mode of 0.125 µg/mL and 99th percentile of 0.5 µg/mL for L2–4/7. The MGIT MIC data from this study agreed with these findings (Table 2). L6 strains were even more susceptible with MICs ≤ 0.016 µg/mL in both studies. L5 MICs were 0.03–0.06 µg/mL in both studies, but we tested a greater number of strains (eight vs just two in the literature), demonstrating that the susceptibility of L5 was more similar to that of L2–4/7 than that of L6. Based on a single replicate for one strain each, it was unclear whether L8 was more similar to L1 or L2–4/7, whereas L9 most resembled L6.

Similar relative susceptibilities of the different lineages were observed on 7H11 (Table 2). For example, the 99th percentile of the L1 distribution at 1 µg/mL was two doubling dilutions higher than for L2–4. MGIT MICs for L1–4 were approximately twice as high as the corresponding 7H11 MICs (Table 3), which was also apparent when comparing the modes of their MIC distributions (Table 2). In contrast, the absolute MICs for L5 and L6 were similar for both media. As only a single strain was tested for L7–8, no meaningful comparison was possible for these lineages.

**TABLE 3:** MGIT to 7H11 MIC ratio for H37Rv and clinical strains without Pa resistance mutations^*a*^

Lineage	No. of strains with their MIC_MGIT_/MIC_7H11_ ratio				
	0.5	1	2	4	8
1	–	2	2	1	–
2	–	–	1	3	–
3	–	–	4	1	–
4	–	–	4	2	1
4-H37Rv	–	1	4	–	–
5	1	5	2	–	–
6	–	7	–	–	–
7	–	1	–	–	–
8	–	–	–	1	–

^*a*^ L9 was excluded as no 7H11 MIC was available, and the ratio was not calculated for strains with at least one truncated MIC (see Table S2 for more details).

## DISCUSSION

This study confirmed that Pa MICs were elevated in L1, regardless of the medium used and phenotype measured [7H11 MIC testing relies on visual growth inhibition on solid medium, whereas MGIT measures oxygen consumption in liquid medium (16)]. The fundamental question for regulators is whether BPaL(M) should be used for L1, even though the clinical outcome data demonstrating good outcomes are more limited for L1 compared with L2–4, therefore, remains pressing in light of the ongoing adoption of this regimen globally (3). In January 2023, the European Committee on Antimicrobial Susceptibility Testing (EUCAST) set a “provisional screen value” of 2 µg/mL for MGIT, which was reaffirmed in 2024, without an accompanying explanation of the meaning or intended use of this concentration (17, 18). Given a history of mistakes when setting breakpoints for MTBC by multiple regulators, we call on EUCAST to publish a justification for its decision to enable external scrutiny (19–22). Moreover, EUCAST should engage with the European Medicines Agency to review its breakpoint for MGIT given that the current choice of 1 mg/L for MGIT is too high for L2–4/7 and low high for L1 (23). WHO reviewed these questions independently and is due to publish its decision shortly.

L6, which causes up to half of TB in some West African countries yet appears underrepresented among rifampicin-resistant strains, was more susceptible than L5 and L2–4/7 and should, therefore, respond better to BPaL(M) (3, 24). More MICs are needed for L8, L9, and the different animal-adapted MTBC genotypes (e.g., *Mycobacterium bovis*), and it would be desirable to clarify their likely response to BPaL(M), although this is not a priority as these are much rarer than L1–7 (3, 14, 24).

MICs for L1–4 appeared to be systematically lower in 7H11 than in MGIT in this single-site study, requiring confirmation in other laboratories [i.e., technical variability may account for this apparent difference, as previously observed for H37Rv tested in different media in MGIT (3, 25–27)].

Our findings further underline the importance of the EUCAST requirements to consider MIC data from multiple laboratories and from phylogenetically diverse MTBC strains (3, 25, 26). Accordingly, the TB Alliance is preparing a study to define the L1 and non-L1 MIC distributions using the EUCAST reference method (26). Commercial phenotypic AST devices (e.g., a lyophilized Pa product for MGIT or a lyophilized broth microdilution assay, which would be preferable to manually weigh Pa for MGIT testing as is the only option currently) will have to be calibrated against the reference method to ensure that any MIC differences are fully systematic (e.g., that the technical variability is not excessive, resulting in wider MIC distributions and, thus, increasing the likelihood of very major diagnostic errors) (26, 28, 29). In the future, such quality-assured and comprehensively validated commercial AST assays should be co-developed with novel relevant antimicrobials given that empiric use risks their long-term utility (30–32).

## SUPPLEMENTAL MATERIAL

Tables S1 and S2 spectrum.00070-24-s0001.docx Overviews of the strains tested.

ASM does not own the copyrights to Supplemental Material that may be linked to, or accessed through, an article. The authors have granted ASM a non-exclusive, world-wide license to publish the Supplemental Material files. Please contact the corresponding author directly for reuse.

## References

[1] World Health Organization. 2023. Global tuberculosis report 2023. Available from: https://iris.who.int/handle/10665/373828

[2] Gupta A, Juneja S, Babawale V, Rustam Majidovich N, Ndjeka N, Thi Mai Nguyen P, Nargiza Nusratovna P, Robert Omanito D, Tiara Pakasi T, Terleeva Y, Toktogonova A, Waheed Y, Myint Z, Yanlin Z, Sahu S. 2024. Global adoption of 6-month drug-resistant TB regimens: projected uptake by 2026. PLoS One 19:e0296448. doi:10.1371/journal.pone.0296448 38180980 PMC10769048

[3] Bateson A, Ortiz Canseco J, McHugh TD, Witney AA, Feuerriegel S, Merker M, Kohl TA, Utpatel C, Niemann S, Andres S, et al.. 2022. Ancient and recent differences in the intrinsic susceptibility of Mycobacterium tuberculosis complex to pretomanid. J Antimicrob Chemother 77:1685–1693. doi:10.1093/jac/dkac070 35260883 PMC9155602

[4] Lee BM, Harold LK, Almeida DV, Afriat-Jurnou L, Aung HL, Forde BM, Hards K, Pidot SJ, Ahmed FH, Mohamed AE, Taylor MC, West NP, Stinear TP, Greening C, Beatson SA, Nuermberger EL, Cook GM, Jackson CJ. 2020. Predicting nitroimidazole antibiotic resistance mutations in Mycobacterium tuberculosis with protein engineering. PLoS Pathog 16:e1008287. doi:10.1371/journal.ppat.1008287 32032366 PMC7032734

[5] Timm J, Bateson A, Solanki P, Paleckyte A, Witney AA, Rofael SAD, Fabiane S, Olugbosi M, McHugh TD, Sun E. 2023. Baseline and acquired resistance to bedaquiline, linezolid and Pretomanid, and impact on treatment outcomes in four tuberculosis clinical trials containing pretomanid. PLOS Glob Public Health 3:e0002283. doi:10.1371/journal.pgph.0002283 37851685 PMC10584172

[6] Battaglia S, Spitaleri A, Cabibbe AM, Meehan CJ, Utpatel C, Ismail N, Tahseen S, Skrahina A, Alikhanova N, Mostofa Kamal SM, Barbova A, Niemann S, Groenheit R, Dean AS, Zignol M, Rigouts L, Cirillo DM. 2020. Characterization of genomic variants associated with resistance to bedaquiline and delamanid in naive Mycobacterium tuberculosis clinical strains. J Clin Microbiol 58:e01304-20. doi:10.1128/JCM.01304-20 32907992 PMC7587096

[7] Merker M, Kohl TA, Barilar I, Andres S, Fowler PW, Chryssanthou E, Ängeby K, Jureen P, Moradigaravand D, Parkhill J, Peacock SJ, Schön T, Maurer FP, Walker T, Köser C, Niemann S. 2020. Phylogenetically informative mutations in genes implicated in antibiotic resistance in Mycobacterium tuberculosis complex. Genome Med 12:27. doi:10.1186/s13073-020-00726-5 32143680 PMC7060619

[8] Kadura S, King N, Nakhoul M, Zhu H, Theron G, Köser CU, Farhat M. 2020. Systematic review of mutations associated with resistance to the new and repurposed Mycobacterium tuberculosis drugs bedaquiline, clofazimine, linezolid, delamanid and pretomanid. J Antimicrob Chemother 75:2031–2043. doi:10.1093/jac/dkaa136 32361756 PMC7825472

[9] Mohamed S, Köser CU, Salfinger M, Sougakoff W, Heysell SK. 2021. Targeted next-generation sequencing: a Swiss army knife for mycobacterial diagnostics? Eur Respir J 57:2004077. doi:10.1183/13993003.04077-2020 33737379 PMC8920038

[10] Abrahams KA, Batt SM, Gurcha SS, Veerapen N, Bashiri G, Besra GS. 2023. DprE2 is a molecular target of the anti-tubercular nitroimidazole compounds pretomanid and delamanid. Nat Commun 14:3828. doi:10.1038/s41467-023-39300-z 37380634 PMC10307805

[11] World Health Organization. 2023. Use of targeted next-generation sequencing to detect drug-resistant tuberculosis: rapid communication. Available from: https://apps.who.int/iris/handle/10665/371687

[12] World Health Organization. 2023. Catalogue of mutations in Mycobacterium tuberculosis complex and their association with drug resistance, second edition. Available from: https://iris.who.int/handle/10665/374061

[13] Coscolla M, Gagneux S, Menardo F, Loiseau C, Ruiz-Rodriguez P, Borrell S, Otchere ID, Asante-Poku A, Asare P, Sánchez-Busó L, et al.. 2021. Phylogenomics of Mycobacterium africanum reveals a new lineage and a complex evolutionary history. Microb Genom 7:000477. doi:10.1099/mgen.0.000477 33555243 PMC8208692

[14] Koleske BN, Jacobs WR, Bishai WR. 2023. The Mycobacterium tuberculosis genome at 25 years: lessons and lingering questions. J Clin Invest 133:e173156. doi:10.1172/JCI173156 37781921 PMC10541200

[15] World Health Organization. 2021. Meeting report of the WHO Expert consultation on the definition of extensively drug-Resistant tuberculosis. 27-29 October 2020. Available from: https://apps.who.int/iris/handle/10665/338776

[16] Pfyffer GE, Palicova F, Rüsch-Gerdes S. 2002. Testing of susceptibility of Mycobacterium tuberculosis to pyrazinamide with the nonradiometric BACTEC MGIT 960 system. J Clin Microbiol 40:1670–1674. doi:10.1128/JCM.40.5.1670-1674.2002 11980940 PMC130957

[17] European Committee on Antimicrobial Susceptibility Testing. 2023. Breakpoint tables for interpretation of Mics and zone diameters. version 13.0, valid from 2023-01-01. Available from: https://www.eucast.org/fileadmin/src/media/PDFs/EUCAST_files/Breakpoint_tables/v_13.0_Breakpoint_Tables.xlsx

[18] European Committee on Antimicrobial Susceptibility Testing. 2024. Breakpoint tables for interpretation of Mics and zone diameters. version 14.0, valid from 2024-01-01. Available from: https://www.eucast.org/fileadmin/src/media/PDFs/EUCAST_files/Breakpoint_tables/v_14.0_Breakpoint_Tables.xlsx

[19] World Health Organization. 2018. Technical report on critical concentrations for drug susceptibility testing of medicines used in the treatment of drug-resistant tuberculosis. Available from: https://apps.who.int/iris/handle/10665/260470

[20] World Health Organization. 2021. Technical report on critical concentrations for drug susceptibility testing of isoniazid and the rifamycins (rifampicin, rifabutin and rifapentine). Available from: https://apps.who.int/iris/handle/10665/339275

[21] Georghiou SB, Rodwell TC, Korobitsyn A, Abbadi SH, Ajbani K, Alffenaar J-W, Alland D, Alvarez N, Andres S, Antimycobacterial Susceptibility Testing Group. 2022. Updating the approaches to define susceptibility and resistance to anti-tuberculosis agents: implications for diagnosis and treatment. Eur Respir J 59:2200166. doi:10.1183/13993003.00166-2022 35422426 PMC9059840

[22] Köser CU, Georghiou SB, Schön T, Salfinger M. 2021. On the consequences of poorly defined breakpoints for rifampin susceptibility testing of Mycobacterium tuberculosis complex. J Clin Microbiol 59:e02328-20. doi:10.1128/JCM.02328-20 33568463 PMC8092724

[23] European Medicines Agency. 2021. Dovprela: EPAR – product information. Available from: https://www.ema.europa.eu/en/documents/product-information/dovprela-epar-product-information_en.pdf

[24] Gagneux S. 2018. Ecology and evolution of Mycobacterium tuberculosis. Nat Rev Microbiol 16:202–213. doi:10.1038/nrmicro.2018.8 29456241

[25] European Committee on Antimicrobial Susceptibility Testing. 2019. SOP for calibrating Surrogate MIC methods for M. tuberculosis against the EUCAST reference MIC method. version 1.0. Available from: http://www.eucast.org/fileadmin/src/media/PDFs/EUCAST_files/Mycobacteria/Methods_in_AMST/CalibrationSOP_Mtb_190718.pdf

[26] Schön T, Köser CU, Werngren J, Viveiros M, Georghiou S, Kahlmeter G, Giske C, Maurer F, Lina G, Turnidge J, van Ingen J, Jankovic M, Goletti D, Cirillo DM, Santin M, Cambau E, ESGMYC. 2020. What is the role of the EUCAST reference method for MIC testing of the Mycobacterium tuberculosis complex? Clin Microbiol Infect 26:1453–1455. doi:10.1016/j.cmi.2020.07.037 32768492

[27] Kahlmeter G, Turnidge J. 2023. Wild-type distributions of minimum inhibitory concentrations and epidemiological cut-off values-laboratory and clinical utility. Clin Microbiol Rev 36:e0010022. doi:10.1128/cmr.00100-22 38038445 PMC10732016

[28] World Health Organization. 2022. Optimized broth microdilution plate methodology for drug susceptibility testing of Mycobacterium tuberculosis complex. Available from: https://apps.who.int/iris/handle/10665/353066

[29] Köser CU, Maurer FP. 2023. Minimum inhibitory concentrations and sequencing data have to be analysed in more detail to set provisional epidemiological cut-off values for Mycobacterium tuberculosis complex. Eur Respir J 61:2202397. doi:10.1183/13993003.02397-2022 37147011

[30] Köser CU, Javid B, Liddell K, Ellington MJ, Feuerriegel S, Niemann S, Brown NM, Burman WJ, Abubakar I, Ismail NA, Moore D, Peacock SJ, Török ME. 2015. Drug-resistance mechanisms and tuberculosis drugs. Lancet 385:305–307. doi:10.1016/S0140-6736(14)62450-8 25706840 PMC4374148

[31] Köser CU, Maurer FP, Kranzer K. 2019. `Those who cannot remember the past are condemned to repeat it´: drug-susceptibility testing for bedaquiline and delamanid. Int J Infect Dis 80S:S32–S35. doi:10.1016/j.ijid.2019.02.027 30818049

[32] Ness T, Van LH, Petermane I, Duarte R, Lange C, Menzies D, Cirillo DM. 2023. Rolling out new anti-tuberculosis drugs without diagnostic capacity. Breathe (Sheff) 19:230084. doi:10.1183/20734735.0084-2023 37492347 PMC10365078

