# Supplementary material for: Refined understanding of the impact of the Mycobacterium tuberculosis complex diversity on the intrinsic susceptibility to pretomanid
Source: Microbiol Spectr. 2024 Feb 9;12(3):e00070-24. doi: 10.1128/spectrum.00070-24 (PMC10913522; doi:10.1128/spectrum.00070-24)
Supplement: Tables S1 and S2 — Overviews of the strains tested. [file spectrum.00070-24-s0001.docx]

**Table S1:** Overview of the 125 MTBC strains without Pa resistance mutations from Table 2 included in this study

| Lineage | Medium | Total tested | Proportion of tested strains | No of strains tested by drug resistance profile | | | | Geographical origin based on WHO regions | | | | | | |
| --- | --- | --- | --- | --- | --- | --- | --- | --- | --- | --- | --- | --- | --- | --- |
|  |  |  |  | DS | Mono/PDR | MDR | Pre-XDR | African | Americas | South-East Asian | European | Eastern-Mediterranean | Western-Pacific |  |
| 1 | 7H11 | 21 | 17% | 11 | 7 | 3 | - | 1 | 0 | 12 | 2 | 0 | 6 |  |
|  | MGIT | 5 | 12% | 5 | - | - | - | 0 | 0 | 2 | 1 | 0 | 2 |  |
| 2 | 7H11 | 30 | 24% | 5 | 12 | 13 | - | 1 | 0 | 10 | 5 | 0 | 14 |  |
|  | MGIT | 4 | 10% | 2 | - | 2 | - | 0 | 0 | 0 | 2 | 0 | 2 |  |
| 3 | 7H11 | 11 | 9% | 9 | 2 | - | - | 4 | 0 | 2 | 3 | 1 | 1 |  |
|  | MGIT | 5 | 12% | 4 | 1 | - | - | 0 | 0 | 2 | 2 | 0 | 1 |  |
| 4 | 7H11 | 38 | 30% | 14 | 12 | 11 | 1 | 13 | 8 | 3 | 8 | 1 | 5 |  |
|  | MGIT | 7 | 17% | 6 | - | 1 | - | 2 | 1 | 1 | 3 | 0 | 0 |  |
| 5 | 7H11 | 11 | 9% | 10 | - | 1 | - | 10 | 1 | 0 | 0 | 0 | 0 |  |
|  | MGIT | 8 | 19% | 7 | - | 1 | - | 7 | 1 | 0 | 0 | 0 | 0 |  |
| 6 | 7H11 | 11 | 8% | 10 | - | 1 | - | 9 | 1 | 0 | 1 | 0 | 0 |  |
|  | MGIT | 9 | 22% | 8 | - | 1 | - | 7 | 1 | 0 | 1 | 0 | 0 |  |
| 7 | 7H11 | 1 | 1% | 1 | - | - | - | 1 | 0 | 0 | 0 | 0 | 0 |  |
|  | MGIT | 1 | 2% | 1 | - | - | - | 1 | 0 | 0 | 0 | 0 | 0 |  |
| 8 | 7H11 | 1 | 1% | - | 1 | - | - | 1 | 0 | 0 | 0 | 0 | 0 |  |
|  | MGIT | 1 | 2% | - | 1 | - | - | 1 | 0 | 0 | 0 | 0 | 0 |  |
| 9 | 7H11 | 1 | 1% | 1 | - | - | - | 1 | 0 | 0 | 0 | 0 | 0 |  |
|  | MGIT | 1 | 2% | 1 | - | - | - | 1 | 0 | 0 | 0 | 0 | 0 |  |

DS= drug-susceptible, MDR= multi-drug resistant, PDR= poly-drug resistant, Pre-XDR= MDR+ resistant to a fluoroquinolone.

**Table S2:** Pa MICs for all strains included in this study

| Strain ID | Genome accession | Lineage | Origin | 7H11 MIC  (µg/mL) | 7H11 MIC batch Nr | MGIT MIC  (µg/mL) | MGIT MIC batch Nr | MIC MGIT/7H11 ratio | BCCM ID | If included in Bateson et al., strain ID used; MGIT MIC (µg/mL) |
| --- | --- | --- | --- | --- | --- | --- | --- | --- | --- | --- |
| 1968-01344 | ERR12115306 | 5.2 | United States | 0.03 | 7 | 0.06 | 1 | 2 | ITM-500032 | - |
| 1973-03608 | ERR12115307 | 5.1.3 | Central African Republic | 0.06 | 8 | 0.06 | 2 | 1 | ITM-500084 | - |
| 1982-08470 | ERR12115308 | 6.1.1 | France | 0.004 | 7 | 0.004 | 1 | 1 | ITM-500120 | - |
| 1991-07531 | ERR12115309 | 5 | Burkina Faso | 0.06 | 7 | 0.03 | 3 | 2 | ITM-500255 | - |
| 1992-08557 | WGS not available | 5 | Cote D'Ivoire | 0.06 | 7 | - | - | - | ITM-500259 | - |
| 1996-00683 | ERR3132183 | 1.1.3 | Bangladesh | 0.5 | 3 | 1 | 1 | 2 | ITM-500321 | - |
| 1996-00723 | ERR3132226 | 1.1.3 | Bangladesh | 0.5 | 4 | - | - | - | ITM-500322 | - |
| 1996-01650 | ERR3132259 | 4.3.4.2 | Bangladesh | 0.03 | 5 | 0.125 | 1 | 4 | ITM-500338 | - |
| 1996-01863 | ERR3132281 | 2.2.1 | Bangladesh | 0.06 | 3 | - | - | - | ITM-500340 | - |
| 1997-00472 | ERR3132292 | 1.1.2 | Bangladesh | 0.75 | 6 | - | - | - | ITM-500342 | - |
| 1997-00991 | ERR3132073 | 2.2.2 | Bangladesh | 0.03 | 6 | - | - | - | ITM-500345 | - |
| 1997-01510 | ERR3132084 | 1.1.2 | Bangladesh | 0.25 | 4 | - | - | - | ITM-500350 | - |
| 1998-00166 | ERR3132106 | 4.4 | Bangladesh | 0.06 | 4 | - | - | - | ITM-500352 | - |
| 1998-00277 | ERR12115310 | 1.1.3.1 | Bangladesh | 0.25 | 3 | - | - | - | ITM-500354 | - |
| 1998-01164 | ERR3132117 | 1.2.2 | Bangladesh | 0.5 | 3 | - | - | - | ITM-500357 | TB-TDR-0014; 2 |
| 1999-00020 | ERR12115311 | 2.2.1 | Russian Fed | 0.125 | 2 | - | - | - | ITM-500364 | - |
| 1999-00168 | ERR3132128 | 2.2.1 | Bangladesh | 0.125 | 3 | - | - | - | ITM-500365 | - |
| 1999-01202 | ERR3132139 | 4.6.1.2 | DR Congo | 0.03 | 5 | 0.125 | 4 | 4 | ITM-500367 | - |
| 1999-01341 | ERR3132172 | 2.2.1 | Azerbaidjan | 0.06 | 2 | 0.25 | 2 | 4 | ITM-500370 | TB-TDR-0019; 0.25 |
| 1999-01545 | ERR3132212 | 4.3.3 | DR Congo | 0.06 | 3 | - | - | - | ITM-500374 | - |
| 1999-01548 | ERR3132220 | 4.6.1.2 | DR Congo | 0.06 | 3 | - | - | - | ITM-500375 | - |
| 1999-01994 | ERR3132221 | 1.2.2 | Bangladesh | 0.5 | 3 | - | - | - | ITM-500378 | - |
| 2000-00092 | ERR12115312 | 1.1.3.2 | Malawi | 0.75 | 6 | - | - | - | ITM-500385 | - |
| 2000-00288 | ERR3132225 | 4.4.1.1 | Azerbaidjan | 0.06 | 2 | - | - | - | ITM-500386 | - |
| 2000-00440 | ERR3132228 | 2.2.1 | Kazakstan | 0.06 | 3 | - | - | - | ITM-500388 | - |
| 2000-00446 | ERR3132229 | 2.2.1 | Bangladesh | 0.06 | 2 | - | - | - | ITM-500389 | TB-TDR-0032; 0.25 |
| 2000-00448 | ERR3132230 | 4.7 | Belgium | 0.03 | 3 | - | - | - | ITM-500390 | - |
| 2000-00611 | ERR3132232 | 2.2.1 | Bangladesh | 0.06 | 4 | - | - | - | ITM-500394 | - |
| 2000-01353 | ERR3132234 | 1.2.2 | Bangladesh | 1 | 6 | - | - | - | ITM-500404 | - |
| 2000-01699 | ERR3132238 | 2.2.1 | Bangladesh | 0.06 | 2 | - | - | - | ITM-500408 | - |
| 2001-00204 | WGS not available | 1 | Bangladesh | 0.5 | 2 | - | - | - | ITM-500414 | - |
| 2001-00294 | ERR3132239 | 3 | Bangladesh | 0.03 | 3 | 0.125 | 1 | 4 | ITM-500416 | - |
| 2001-00299 | ERR3132240 | 1.1.2 | Bangladesh | 1 | 6 | - | - | - | ITM-500417 | - |
| 2001-00966 | ERR3132242 | 2.2.1 | Bangladesh | 0.06 | 2 | - | - | - | ITM-500432 | - |
| 2001-01466 | WGS not available | 1 | Bangladesh | 0.75 | 5 | - | - | - | ITM-500447 | - |
| 2002-02478 | ERR12115313 | 2.2.1 | Bangladesh | 0.06 | 2 | - | - | - | ITM-500477 | - |
| 2003-01714 | ERR3132257 | 4.6.1.2 | Burundi | 0.125 | 2 | - | - | - | ITM-500488 | - |
| 2003-01801 | ERR3132260 | 4.3.4.2.1 | Burundi | 0.06 | 4 | - | - | - | ITM-500490 | - |
| 2004-00851 | ERR3132266 | 4.1.1.3 | South Africa | 0.06 | 5 | - | - | - | ITM-500504 | - |
| 2004-01086 | ERR3132089 | 4.6.1.2 | Rwanda | 0.125 | 4 | - | - | - | ITM-500506 | - |
| 2004-01195 | ERR3132279 | 2.2.1 | South Korea | 0.06 | 3 | 0.25 | 2 | 4 | ITM-500517 | - |
| 2004-01198 | ERR3132283 | 2.2.1 | South Korea | 0.06 | 3 | - | - | - | ITM-500520 | - |
| 2004-01199 | ERR3132284 | 2.2.1 | South Korea | 0.06 | 3 | - | - | - | ITM-500521 | - |
| 2004-01200 | ERR3132285 | 3 | South Korea | 0.06 | 3 | 0.125 | 2 | 2 | ITM-500522 | - |
| 2004-01202 | ERR3132286 | 2.2.1 | South Korea | 0.125 | 4 | - | - | - | ITM-500523 | - |
| 2004-01203 | ERR3132287 | 2.2.1 | South Korea | 0.06 | 4 | - | - | - | ITM-500524 | - |
| 2004-01204 | ERR3132288 | 2.2.1 | South Korea | 0.06 | 3 | - | - | - | ITM-500525 | - |
| 2004-01205 | ERR3132289 | 4.9 | South Korea | 0.125 | 2 | - | - | - | ITM-500526 | - |
| 2004-01207 | ERR3132291 | 4.5 | South Korea | 0.03 | 5 | - | - | - | ITM-500528 | - |
| 2004-01212 | ERR3132296 | 4 | South Korea | 0.03 | 4 | - | - | - | ITM-500532 | - |
| 2004-01219 | ERR3132302 | 2.2.2 | South Korea | 0.06 | 3 | - | - | - | ITM-500538 | - |
| 2004-01220 | ERR3132074 | 2.2.2 | South Korea | 0.06 | 2 | - | - | - | ITM-500539 | - |
| 2004-01237 | ERR3132268 | 2.2.2 | South Korea | 0.03 | 3 | - | - | - | ITM-500550 | - |
| 2004-01239 | ERR3132087 | 2.2.2 | South Korea | 0.06 | 4 | - | - | - | ITM-500551 | - |
| 2004-01241 | ERR3132112 | 2.2.1 | South Korea | 0.06 | 2 | - | - | - | ITM-500553 | - |
| 2004-01244 | ERR3132092 | 2.2.2 | South Korea | 0.06 | 4 | - | - | - | ITM-500556 | - |
| 2004-01248 | ERR3132094 | 4 | South Korea | 0.06 | 4 | - | - | - | ITM-500558 | - |
| 2004-01259 | ERR3132100 | 2.2.2 | South Korea | 0.25 | 4 | - | - | - | ITM-500563 | - |
| 2004-01285 | ERR3132105 | 4 | Brazil | 0.06 | 4 | - | - | - | ITM-500568 | - |
| 2004-01286 | ERR3132107 | 4.3.4.2 | Brazil | 0.03 | 5 | 0.25 | 2 | 8 | ITM-500569 | - |
| 2004-01289 | ERR3132108 | 4.3.4.2 | Brazil | 0.03 | 4 | - | - | - | ITM-500570 | - |
| 2004-01458 | ERR3132111 | 4.3.4.2.1 | Rwanda | 0.06 | 4 | - | - | - | ITM-500574 | - |
| 2004-01659 | ERR3132116 | 4.6.1.1 | Germany | 0.03 | 2 | - | - | - | ITM-500582 | - |
| 2004-01670 | ERR3132123 | 4.8 | Ukraine | 0.03 | 4 | - | - | - | ITM-500589 | - |
| 2004-01679 | ERR3132126 | 2.2.1 | Nepal | 0.25 | 4 | - | - | - | ITM-500592 | - |
| 2004-02160 | ERR3132142 | 4.6.1.2 | Rwanda | 0.125 | 3 | - | - | - | ITM-500599 | - |
| 2004-02613 | ERR3132151 | 4.1.2.1 | Peru | 0.03 | 4 | - | - | - | ITM-500618 | - |
| 2004-02614 | ERR3132152 | 4 | Peru | 0.25 | 4 | - | - | - | ITM-500619 | - |
| 2004-02760 | ERR3132154 | 1.2.1 | Philippines | 0.25 | 4 | - | - | - | ITM-500621 | - |
| 2004-02763 | ERR3132156 | 1.2.1 | Philippines | 0.5 | 3 | - | - | - | ITM-500623 | - |
| 2004-02765 | ERR3132157 | 1.2.1 | Philippines | 0.75 | 6 | - | - | - | ITM-500624 | - |
| 2004-02911 | ERR3132162 | 4.1.2 | Germany | 0.03 | 2 | - | - | - | ITM-500630 | - |
| 2004-02915 | ERR3132166 | 3 | Pakistan | 0.06 | 4 | 0.125 | 1 | 2 | ITM-500634 | - |
| 2004-02916 | ERR3132167 | 4.6.2.2 | Nigeria | 0.03 | 5 | - | - | - | ITM-500635 | - |
| 2004-02921 | ERR3132171 | 1.1.2 | China (Tibet) | 0.5 | 3 | - | - | - | ITM-500639 | TB-TDR-0189; 1 |
| 2004-02924 | ERR3132174 | 4.3 | Peru | 0.06 | 3 | - | - | - | ITM-500641 | - |
| 2004-02926 | ERR3132176 | 4.3.4.2 | Portugal | 0.03 | 6 | - | - | - | ITM-500643 | - |
| 2004-02927 | ERR3132177 | 4.3.4.2 | Rep. Domin. | 0.06 | 5 | - | - | - | ITM-500644 | - |
| 2005-00691 | ERR3132186 | 4.3 | Peru | 0.03 | 3 | - | - | - | ITM-500652 | - |
| 2005-00706 | ERR3132200 | 4.1.2.1 | Peru | 0.03 | 2 | - | - | - | ITM-500665 | - |
| 2005-02460 | ERR439931 | 4.1.2.1 | Cameroon | 0.06 | 3 | 0.125 | 2 | 2 | ITM-501055 | - |
| 2006-00789 | ERR439937 | 5.1.2 | Benin | 0.03 | 2 | - | - | - | ITM-501069 | - |
| 2006-00790 | ERR439938 | 6.2.1 | Benin | ≤0.002 | 6 | ≤0.002 | 3 | NC | ITM-501070 | - |
| 2006-00795 | ERR439941 | 5.1.4 | Benin | 0.06 | 7 | 0.06 | 3 | 1 | ITM-500691 | - |
| 2006-00800 | ERR439945 | 5.3 | Benin | 0.03 | 7 | 0.03 | 1 | 1 | ITM-500692 | - |
| 2006-02410 | ERR12115314 | 5.1.1 | Guinea | 0.06 | 7 | 0.06 | 3 | 1 | ITM-500699 | - |
| 2006-02732 | ERR3132210 | 2.2.1 | Georgia | 0.125 | 4 | - | - | - | ITM-500701 | - |
| 2006-02951 | ERR12115315 | 3.1.1 | Zambia | 0.06 | 6 | - | - | - | ITM-500703 | - |
| 2008-00618 | WGS not available | 6 | Senegal | 0.004 | 7 | 0.004 | 1 | 1 | ITM-500722 | - |
| 2008-01456 | ERR439971 | 6.3.1 | Guinea | 0.004 | 6 | 0.004 | 1 | 1 | ITM-501058 | - |
| 2008-04431 | ERR3132213 | 2.2.1 | DR Congo | 0.06 | 3 | - | - | - | ITM-500737 | - |
| 2009-02073 | ERR3132215 | 4.6.2.2 | Niger | 0.03 | 3 | - | - | - | ITM-500745 | - |
| 2010-01231 | WGS not available | 6 | Senegal | 0.004 | 6 | 0.004 | 3 | 1 | ITM-500758 | - |
| 2011-02171 | ERR12115303 | 6 | Gambia | 0.004 | 6 | 0.004 | 1 | 1 | ITM-500782 | - |
| 2012-00445 | ERR12115316 | 3 | Sweden | 0.125 | 7 | - | - | - | ITM-500785 | - |
| 2012-00752 | ERR12115317 | 5.1.2 | Niger | 0.06 | 7 | 0.06 | 2 | 1 | ITM-501059 | - |
| 2013-00036 | ERR12115318 | 6.1.1 | United States | 0.004 | 7 | 0.004 | 2 | 1 | ITM-500811 | - |
| 2013-02158 | ERR12115319 | 3.1.1 | Kenya | 0.06 | 7 | - | - | - | ITM-500854 | - |
| 2013-02481 | ERR8025345 | 1.1.3 | Belgium | >8 | 6 | >4 | - | - | ITM-500859 | ITM 500859; >16 |
| 2013-03157 | WGS not available | 6 | Gambia | 0.004 | 7 | 0.004 | 3 | 1 | ITM-500873 | - |
| 2013-03158 | WGS not available | 6 | Gambia | 0.004 | 7 | - | - | - | ITM-500874 | - |
| 2014-01606 | ERR12115320 | 1.2.2.2 | Belgium | 0.75 | 6 | - | - | - | ITM-500884 | - |
| 2018-00082 | ERR2704704; ERR2704685 | 1.2.1 | Philippines | 0.75 | 6 | 1 | 3 | 1 | ITM-500941 | - |
| 2018-00083 | ERR2704680 | 1.1.2 | India | 0.5 | 2 | 1 | 2 | 2 | ITM-500942 | - |
| 2018-00089 | ERR2704675; ERR2704697; ERR2704696 | 3.1 | India | 0.06 | 4 | 0.125 | 3 | 2 | ITM-500947 | - |
| 2018-00090 | ERR2704693 | 3.1.3 | Afghanistan | 0.125 | 3 | - | - | - | ITM-500948 | - |
| 2018-00091 | ERR2704678 | 3 | Ethiopia | 0.125 | 2 | - | - | - | ITM-500949 | - |
| 2018-00092 | ERR2704705; ERR2704689 | 4.6.2.2 | Ghana | 0.06 | 2 | - | - | - | ITM-500950 | - |
| 2018-00095 | ERR2704686 | 5.1.1 | Ghana | 0.125 | 2 | 0.06 | 3 | 0.5 | ITM-500953 | - |
| 2018-00097 | ERR2704706; ERR2704690 | 5.1 | Sierra Leone | 0.03 | 2 | - | - | - | ITM-500955 | - |
| 2018-00098 | ERR2704687 | 6.3.1 | Ghana | invalid | 2 | 0.008 | 5 | NC | ITM-500956 | - |
| 2018-00099 | ERR2704681 | 6.1.1 | Gambia | 0.004 | 8 | - | - | - | ITM-500957 | - |
| 2018-00101 | ERR2704711; ERR2704695; ERR2704710 | 7 | Ethiopia | 0.125 | 5 | 0.125 | 3 | 1 | ITM-500959 | 12195/18; 0.25 |
| 2018-00102 | ERR2704679; ERR2704700 | 1.1.1 | China | 0.5 | 2 | 0.5 | 2 | 1 | ITM-500960 | - |
| 2018-01172 | ERR12115321 | 8 | Rwanda | 0.25 | 2 | 1 | 5 | 4 | ITM-500961 | - |
| 2018-03241 | ERR2704677; ERR2704699; ERR270469 | 2.2.2 | China | 0.06 | 2 | 0.125 | 2 | 2 | ITM-500976 | - |
| 2019-03960 | ERR12115322 | 3.1.1 | Tanzania | 0.03 | 2 | - | - | - | ITM-501067 | - |
| 2020-03563 | ERR7361924 | 1 | United Kingdom | 0.25 | 1 | 1 | 5 | 4 | ITM-501090 | - |
| 2020-03564 | ERR7361925 | 3 | United Kingdom | 0.06 | 1 | 0.125 | 4 | 2 | ITM-501091 | - |
| 2020-03565 | SAMN11179707 | 1.1.3 | United Kingdom | >8 | 6 | >4 | 5 | NC | ITM-501092 | PRE_004, (TN34503), ITM 501092; >8 |
| 2020-03567 | ERR7361927 | 4 | United Kingdom | 0.06 | 1 | 0.125 | 5 | 2 | ITM-501094 | - |
| 2020-03568 | ERR7361928 | 2.2.1 | Ukraine | >8 | 8 | >4 | 5 | NC | ITM-501095 | 14A1; >16 |
| 2020-03572 | SAMN11179714 | 4.8 | United Kingdom | 0.125 | 1 | 0.25 | 4 | 2 | ITM-501099 | PRE_012 (TN33547), ITM 501099; 0.5 |
| 2021-00504 | ERR12115323 | 9 | Rwanda | invalid | 8 | ≤0.002 | 5 | NC | ITM-501138 | - |
| 2020-03571 | SAMN11179698 | 2.2.1 | United Kingdom | 0.06 | 8 | 0.25 | 5 | 4 | ITM-501098 | PRE_011 (TN11265), ITM 501098; 0.25 |
| 2020-03570 | ERR7361929 | 4.3.3 | United Kingdom | 0.06 | 8 | 0.125 | 4 | 2 | ITM-501097 | - |
| 2020-00018 | ERR12115305 | 2 | India | 0.125 | 8 | - | - | - | NA | - |
| 2020-00011 | ERR12115304 | 1.1.3 | Bangladesh | >8 | 4 | >4 | 2 | NC | NA | - |
| 1996-01852 | ERR3132270 | 4 | Bangladesh | 0.03 | 3 | - | - | - | ITM-500339 | - |
| H37Rv | - | 4 | BCCM/ITM | 0.06 | 1 | 0.125 | 1 | 2 | ITM-500735 | - |
| H37Rv | - | 4 | BCCM/ITM | 0.125 | 2 | 0.125 | 2 | 1 | ITM-500735 | - |
| H37Rv | - | 4 | BCCM/ITM | 0.125 | 3 | 0.25 | 3 | 2 | ITM-500735 | - |
| H37Rv | - | 4 | BCCM/ITM | 0.125 | 4 | 0.25 | 4 | 2 | ITM-500735 | - |
| H37Rv | - | 4 | BCCM/ITM | 0.125 | 5 | 0.25 | 5 | 2 | ITM-500735 | - |
| H37Rv | - | 4 | BCCM/ITM | 0.125 | 6 | - | - | - | ITM-500735 | - |
| H37Rv | - | 4 | BCCM/ITM | 0.06 | 7 | - | - | - | ITM-500735 | - |
| H37Rv | - | 4 | BCCM/ITM | 0.125 | 8 | - | - | - | ITM-500735 | - |
| H37Rv | - | 4 | BCCM/ITM | 0.125 | QC Range-W1.B1 | - | - | - | ITM-500735 | - |
| H37Rv | - | 4 | BCCM/ITM | 0.125 | QC Range-W1.B2 | - | - | - | ITM-500735 | - |
| H37Rv | - | 4 | BCCM/ITM | 0.125 | QC Range-W1.B3 | - | - | - | ITM-500735 | - |
| H37Rv | - | 4 | BCCM/ITM | 0.125 | QC Range-W2.B1 | - | - | - | ITM-500735 | - |
| H37Rv | - | 4 | BCCM/ITM | 0.125 | QC Range-W2.B2 | - | - | - | ITM-500735 | - |
| H37Rv | - | 4 | BCCM/ITM | 0.125 | QC Range-W2.B3 | - | - | - | ITM-500735 | - |
| H37Rv | - | 4 | BCCM/ITM | 0.125 | QC Range-W3.B1 | - | - | - | ITM-500735 | - |
| H37Rv | - | 4 | BCCM/ITM | 0.125 | QC Range-W3.B2 | - | - | - | ITM-500735 | - |
| H37Rv | - | 4 | BCCM/ITM | 0.125 | QC Range-W3.B3 | - | - | - | ITM-500735 | - |
| H37Rv | - | 4 | BCCM/ITM | 0.125 | QC Range-W4.B1 | - | - | - | ITM-500735 | - |
| H37Rv | - | 4 | BCCM/ITM | 0.125 | QC Range-W4.B2 | - | - | - | ITM-500735 | - |
| H37Rv | - | 4 | BCCM/ITM | 0.125 | QC Range-W4.B3 | - | - | - | ITM-500735 | - |
| H37Rv | - | 4 | BCCM/ITM | 0.125 | QC Range-W5.B1 | - | - | - | ITM-500735 | - |
| H37Rv | - | 4 | BCCM/ITM | 0.125 | QC Range-W5.B2 | - | - | - | ITM-500735 | - |
| H37Rv | - | 4 | BCCM/ITM | 0.125 | QC Range-W5.B3 | - | - | - | ITM-500735 | - |
| H37Rv | - | 4 | BCCM/ITM | 0.125 | QC Range-W6.B1 | - | - | - | ITM-500735 | - |
| H37Rv | - | 4 | BCCM/ITM | 0.125 | QC Range-W6.B2 | - | - | - | ITM-500735 | - |
| H37Rv | - | 4 | BCCM/ITM | 0.125 | QC Range-W6.B3 | - | - | - | ITM-500735 | - |
| H37Rv | - | 4 | BCCM/ITM | 0.125 | QC Range-W7.B1 | - | - | - | ITM-500735 | - |
| H37Rv | - | 4 | BCCM/ITM | 0.125 | QC Range-W7.B2 | - | - | - | ITM-500735 | - |
| H37Rv | - | 4 | BCCM/ITM | 0.125 | QC Range-W7.B3 | - | - | - | ITM-500735 | - |
| H37Rv | - | 4 | BCCM/ITM | 0.125 | QC Range-W8.B1 | - | - | - | ITM-500735 | - |
| H37Rv | - | 4 | BCCM/ITM | 0.125 | QC Range-W8.B2 | - | - | - | ITM-500735 | - |
| H37Rv | - | 4 | BCCM/ITM | 0.125 | QC Range-W8.B3 | - | - | - | ITM-500735 | - |
| H37Rv | - | 4 | BCCM/ITM | 0.125 | QC Range-W9.B1 | - | - | - | ITM-500735 | - |
| H37Rv | - | 4 | BCCM/ITM | 0.125 | QC Range-W9.B2 | - | - | - | ITM-500735 | - |
| H37Rv | - | 4 | BCCM/ITM | 0.125 | QC Range-W9.B3 | - | - | - | ITM-500735 | - |
| H37Rv | - | 4 | BCCM/ITM | 0.125 | QC Range-W10.B1 | - | - | - | ITM-500735 | - |
| H37Rv | - | 4 | BCCM/ITM | 0.125 | QC Range-W10.B2 | - | - | - | ITM-500735 | - |
| H37Rv | - | 4 | BCCM/ITM | 0.125 | QC Range-W10.B3 | - | - | - | ITM-500735 | - |

NC= Not calculated due to truncated/invalid MIC for at least on one medium, W = Week, B= 7H11-Pa batch nr.

Lineage was determined using WGS data for strains with WGS data and Spoligotyping for strains without WGS data. All strains with a BCCM ID can be purchased from the BCCM/ITM Mycobacteria Collection (https://bccm.belspo.be/about-us/bccm-itm).

To measure the technical variability of MIC testing, 30 replicates of H37Rv (BCCM/ITM, ITM 500735/CT2008-03715) were tested weekly for ten weeks using three different batches of 7H11-Pa media.
